# Rates and predictors of recurrent work disability due to common mental health disorders in the United States

**DOI:** 10.1371/journal.pone.0205170

**Published:** 2018-10-09

**Authors:** Fraser W. Gaspar, Catherine S. Zaidel, Carolyn S. Dewa

**Affiliations:** 1 MDGuidelines, ReedGroup, Ltd. Westminster, Colorado, United States of America; 2 Department of Psychiatry and Behavioral Sciences, University of California at Davis, Davis, California, United States of America; North Carolina Neuropsychiatry Clinics, UNITED STATES

## Abstract

**Context:**

Despite the high prevalence of work disability due to common mental disorders (CMD), no information exists on the rates and predictors of recurrence in a United States population.

**Objective:**

To estimate recurrent work disability statistics and evaluate factors associated with recurrence due to CMDs including adjustment, anxiety, bipolar, and depressive disorders.

**Methods:**

Recurrent work disability statistics were calculated using a nationwide database of disability claims. For the CMDs, univariate and multiple variable analyses were used to examine demographic factors and comorbidities associated with the time to recurrence.

**Results:**

Of the CMDs, cases with bipolar (n = 3,017) and depressive disorders (n = 20,058) had the highest recurrence densities, 98.7 and 70.9 per 1000 person-years, respectively. These rates were more than three times higher than recurrence rates for other chronic disorders (e.g., diabetes, asthma; n = 105,558) and non-chronic disorders (e.g., injury, acute illnesses; n = 153,786). Individuals with CMD were also more likely to have a subsequent disability distinct from their mental health condition. Risk factors for recurrent CMD disability included being younger, being an hourly employee, living in a geographic area with more college graduates, having more previous psychiatric visits, having a previous work leave, and the type of work industry.

**Conclusions:**

Results indicate that CMD patients may benefit from additional care and disability management both during and after their work absence to help prevent subsequent CMD and non-CMD related leaves.

## Introduction

Mental health conditions are one of the leading causes of disability in the United States.[1] The economic impact of mental health in the United States has been estimated to be $467 billion per year.[2] By healthcare expenditure, mental disorders are the most costly condition in the United States.[3] Individuals with mental health conditions may need to take a medically-verified sickness absence, which is a temporary disability leave that pays a portion of their salary, which allows the employee time to recover but reduces income for the employee and productivity for the employer.

While preventing initial disability leaves are important, mental health conditions are often chronic and leave recurrence is common.[4–9] Individuals returning from mental health-related absences are more likely to go back out on leave than individuals with leaves for other disorders/injuries.[6,10] For example, in a study from the Netherlands on employees who worked for the Dutch Post and Telecom, Roelen et al. (2010) found that individuals with a sickness absence due to mental and behavioral disorders had the second highest rate of recurrence by diagnostic group, with only musculoskeletal system disorders having a higher recurrence density.[6] Despite the high prevalence in the United States of common mental health disorders (CMD), which includes adjustment, anxiety, bipolar, and depressive disorders, no study to date has examined disability recurrence statistics.

Understanding the determinants and risk factors for recurrent disability would help to develop effective strategies to reduce recurrent disability due to CMD.[11] Studies on determinants of recurrence are limited and the determinants for each CMD have not been evaluated separately.[6,8,12–14] Arends et al. (2014) observed no relationship between age and recurrence but found an increased risk of recurrent disability for employees that worked in larger companies, had conflicts with a supervisor and did not have any comorbid chronic diseases. [8] However, no study has examined how the association between health care use and prior comorbidities influences work disability recurrence.

In this study, a nationwide database of United States work disability leaves was used to calculate recurrence statistics and identify predictors of recurrent work disability for CMDs.

## Methods

### Data

This study analyzed anonymized data from IBM’s MarketScan Health and Productivity Management and Commercial Claims and Encounters databases using records from 2007 to 2014. The Health and Productivity Management database contains work disability information, including the primary diagnosis (defined using ICD-9-CM) and disability first absence and return-to-work dates. The Commercial Claims and Encounters database contains additional demographic information and healthcare utilization records for the leave cases. All records within the MarketScan databases are linked by a unique identifier. The data contributed to the MarketScan databases come from more than 260 employers and 40 contributing health plans, representing 350 unique carriers.[15]

### Defining index and recurrent leaves

Index leaves were defined as the first work disability with a CMD as the primary diagnosis. For individuals who never experienced a CMD-related leave, the index leave was defined as the first disability episode for a chronic condition. The index leave for individuals without either a CMD or chronic condition was defined as the first mention of a non-chronic condition/injury. The Healthcare Cost and Utilization Project’s Chronic Condition Indicator was used to determine if a condition was considered chronic.[16] The index leave absence dates had to occur between 2008–2013, and the individual had to be eligible for short-term disability benefits in the year prior to the index leave’s start date and the year after the index leave’s return-to-work date. The median follow-up time prior to the index leave was 2.7 years. If an individual immediately transferred to long-term disability, the end of the long-term disability leave was used as the index leave’s end date was. A recurrent leave was defined as a subsequent leave at any point after the index within the follow-up period. Disability durations for index and recurrent leaves were calculated as the number of calendar days between the first absence and return to work dates.

The Healthcare Cost and Utilization Project’s multi-level Clinical Classification Software (CCS) was used to cluster the ICD-9-CM codes into related mental health conditions.[17] The CCS consists of 285 diagnosis categories that collapses individual ICD-9-CM codes into four hierarchical levels. The CCS groupings for adjustment, anxiety, bipolar, and depressive disorders were considered in this analysis. The CCS grouper was used instead of exact ICD-9-CM codes due to the variability in medical codes used for billing, the variability in diagnoses provided by physicians, and the dependent relationships between codes. Examples of non-CMD, chronic condition CCS groups include leukemia, cystic fibrosis, and sickle cell anemia. Examples of non-chronic condition CCS groups include intestinal infections, pneumonia, and lumbago. The ICD-9-CM codes in each group are provided in the Supporting Information (S1 Table).

Person-years of follow-up was calculated as the length of time the employee remained eligible for short-term disability benefits following the end of the index leave. Eligibility for disability benefits is recorded by year in the Health and Productivity Management database; therefore, start and end dates for eligibility were January 1^st^ and December 31^st^. Time spent on any disability leave was not counted in follow-up time calculations, regardless of whether the diagnosis was related to the index leave’s diagnosis or the disability leave was supported under long-term disability or worker’s compensation benefits. The median follow-up time in this study was 2.3 years.

The following work disability recurrence statistics were developed: Probability of recurrence within one year = the probability of a recurrent leave within one calendar year after the index leave (%); Recurrence density = the number of recurrent leaves per 1000 person-years of follow-up (# events/1000 person-years); Days to recurrence = the median number of calendar days between end of the index leave and the start of the first recurrent leave; Recurrence/Index Duration Ratio = the median ratio of disability days for all recurrences (median if multiple) to disability days of the index leave.

### Demographic and disability characteristics

The following variables were abstracted from the IBM’s MarketScan databases: age, sex, geographic location, employee’s job industry (specified by IBM but generally follow the Standard Industrial Classification system), employee’s insurance plan, whether the employee was salaried or in a union, whether the employee experienced an inpatient stay during their index leave, the number of outpatient psychiatric visits in the year prior to index leave, previous work leave, and the presence of a comorbid chronic condition in the year prior to the index leave.

Psychiatric visits included psychiatric diagnostic services, psychotherapy (individual, family, or group), psychiatric advice, and therapeutic psychiatric services. Comorbidities were defined using the ICD-9-CM diagnosis groupings in Quan et al. (2005).[18] Geographically-derived variables were generated for each record by linking the Metropolitan Statistical Area or, if unavailable, the county with data from the 2007–2011 American Community Survey to get the five-year average values for median household income, population density (number of people per square mile), and percent of residents with at least a college degree. These data were abstracted from the Census application program interface with the *ACS* package in R.[19]

### Statistical analyses

Survival models were used to account for the right-censored nature of the data. To visually inspect the differences in recurrence rates by disorder group, Kaplan-Meier curves were generated using the *survminer* package in R.[20] Chi-squared p-values from log-rank tests were used to assess the statistical significance between disorder groups.

For both the univariate and multiple variable predictor analyses, Cox proportional hazard models were used to assess factors associated with time to recurrence. Predictors with the same value for more than 95% of the population were not considered. Age, disability days, median household income, percent with a college education, population density, previous leave and number of outpatient visits were scaled by subtracting the mean and dividing by the standard deviation to allow for easier comparisons of the effect sizes (hazard ratios). Industry variables were dichotomized to compare working in one industry to the other industries combined. Missing data (<18% per variable) were randomly imputed using the empirical distribution.

For the multiple variable regression, a bootstrap backwards selection procedure was used to select significant variables. For each CMD, 100 bootstrap samples were generated, and a backwards variable selection procedure was used to find predictors that were statistically associated (p-value < 0.05) with the outcomes in at least 75% of the bootstrap samples.[21] Significant variables were then fit to the full dataset to evaluate the relationship of predictors with time to recurrence.

By CMD, the proportional hazard assumption was checked for each variable by plotting the scaled Schoenfeld residuals against time. Most p-values from the chi-squared tests of the scaled Schoenfeld residuals were not significant (88%). When significant, the Spearman’s correlations between the residuals and time ranged from -0.08 to 0.09. The only exception was that age for adjustment disorders appeared to violate the proportional hazard model with a rho of -0.36. Overall, the results suggest only minor violations of the proportional hazard assumption, judged unlikely to influence the overall findings.

Univariate analyses were performed on datasets stratified by sex to evaluate whether sex was an effect modifier on the relationship between predictors and recurrence rates. In addition, our bootstrap backwards selection procedure included interaction variables between sex and the other potential predictors.

The representativeness of our study population was evaluated by comparing demographic information to the source and target populations (Table A in S1 File). The source population was defined as the entire MarketScan population with employer-sponsored health and disability insurance coverage, including individuals in the study population. The target population was defined as all employed individuals with employer-sponsored health and disability insurance coverage, based on external data sources. For the target population, it was assumed that individuals with employer-sponsored health plans also were covered by short-term disability benefits, as was the case in the MarketScan database.

Given the observed differences between the characteristics of the study population with both the source and target population, two sets of inverse probability weights (IPW) were generated to standardize the study’s disability recurrence statistics to the source and target populations.[22] To standardize the study population to the source population, IPWs were generated by fitting a logistic regression model to the source population with the dependent variable defined as whether the individual was included in the study (Y = 1 if in the study population, Y = 0 if in the source but not study population). Independent variables included age, sex, health plan type, employer’s industry, whether salaried or union, median household income, percent with a college education, and population density. To standardize the study population to the target population, IPWs were generated using the raking procedures as described by DeBell and Krosnick (2009)[23], implemented using the *anesrake* function in R.[24] Factors used for the raking procedure included age, sex, health plan type, employer’s industry, and whether the employee was in a union (Table B in S1 File). Weights were only used in the calculation of work disability recurrence statistics for the source and target population, not for the analysis of predictors, as representativeness is most important when calculating descriptive statistics.[25] All analyses were performed in R version 3.3.1.[26]

## Results

### Demographics

The final sample included 37,140 cases with CMD, 105,558 cases with another chronic disorder, and 153,786 with an injury or other non-chronic disorder (Table 1). Across all conditions (n = 296,484), there were slightly more males (51.3%) than females and the median age was 45 years. Compared to cases with another chronic disorder or injury/non-chronic disorder, CMD cases were more likely to be female, younger, and hourly employees. Further, CMD cases were more likely to work in the Finance, Insurance, Real Estate, Transportation, Communications, or Utilities sectors.

**Table 1:** Demographic variables of the study population.

	CMD, n = 37,140	Other Chronic, n = 105,558	Injury/ Non-Chronic, n = 153,786	All, n = 296,484
**Sex**				
Female	24,646 (66.4%)	50,603 (47.9%)	69,135 (45%)	144,384 (48.7%)
Male	12,494 (33.6%)	54,955 (52.1%)	84,651 (55%)	152,100 (51.3%)
**Industry**^**a**^				
Construction	3 (<0.1%)	33 (<0.1%)	101 (0.1%)	137 (<0.1%)
Finance, Insurance, Real Estate	11,326 (30.5%)	17,596 (16.7%)	28,989 (18.9%)	57,911 (19.5%)
Manufacturing, Durable Goods	7,887 (21.2%)	35,830 (33.9%)	45,679 (29.7%)	89,396 (30.2%)
Manufacturing, Nondurable Goods	2,676 (7.2%)	13,404 (12.7%)	21,659 (14.1%)	37,739 (12.7%)
Oil & Gas Extraction, Mining	11 (<0.1%)	124 (0.1%)	225 (0.1%)	360 (0.1%)
Retail Trade	986 (2.7%)	4,995 (4.7%)	6,935 (4.5%)	12,916 (4.4%)
Services	1,138 (3.1%)	5,031 (4.8%)	7,499 (4.9%)	13,668 (4.6%)
Transportation, Communications, Utilities	13,113 (35.3%)	28,545 (27%)	42,682 (27.8%)	84,340 (28.4%)
Wholesale	0 (0%)	0 (0%)	14 (0%)	14 (0%)
Unknown	0 (0%)	0 (0%)	3 (0%)	3 (0%)
**Salaried**^**a**^				
No	24,146 (65%)	61,944 (58.7%)	91,683 (59.6%)	177,773 (60%)
Yes	7,515 (20.2%)	28,887 (27.4%)	39,002 (25.4%)	75,404 (25.4%)
Unknown	5,479 (14.8%)	14,727 (14%)	23,101 (15%)	43,307 (14.6%)
**Union**^**a**^				
No	24,351 (65.6%)	70,919 (67.2%)	106,608 (69.3%)	201,878 (68.1%)
Yes	10,508 (28.3%)	31,013 (29.4%)	41,346 (26.9%)	82,867 (27.9%)
Unknown	2,281 (6.1%)	3,626 (3.4%)	5,832 (3.8%)	11,739 (4%)
**Index duration (days)**				
Median (interquartile range)	45 (27–82)	43 (25–74)	34 (19–58)	39 (21–67)
**Follow up time (years)**				
Median (interquartile range)	2.01 (1.36–3.06)	2.22 (1.49–3.31)	2.38 (1.57–3.52)	2.27 (1.51–3.39)
**Age (years)**^**a**^				
Median (interquartile range)	40 (32–48)	49 (40–55)	45 (35–52)	45 (36–53)

^a^ At the time of index leave

### Work disability recurrence statistics

**Fig 1:**
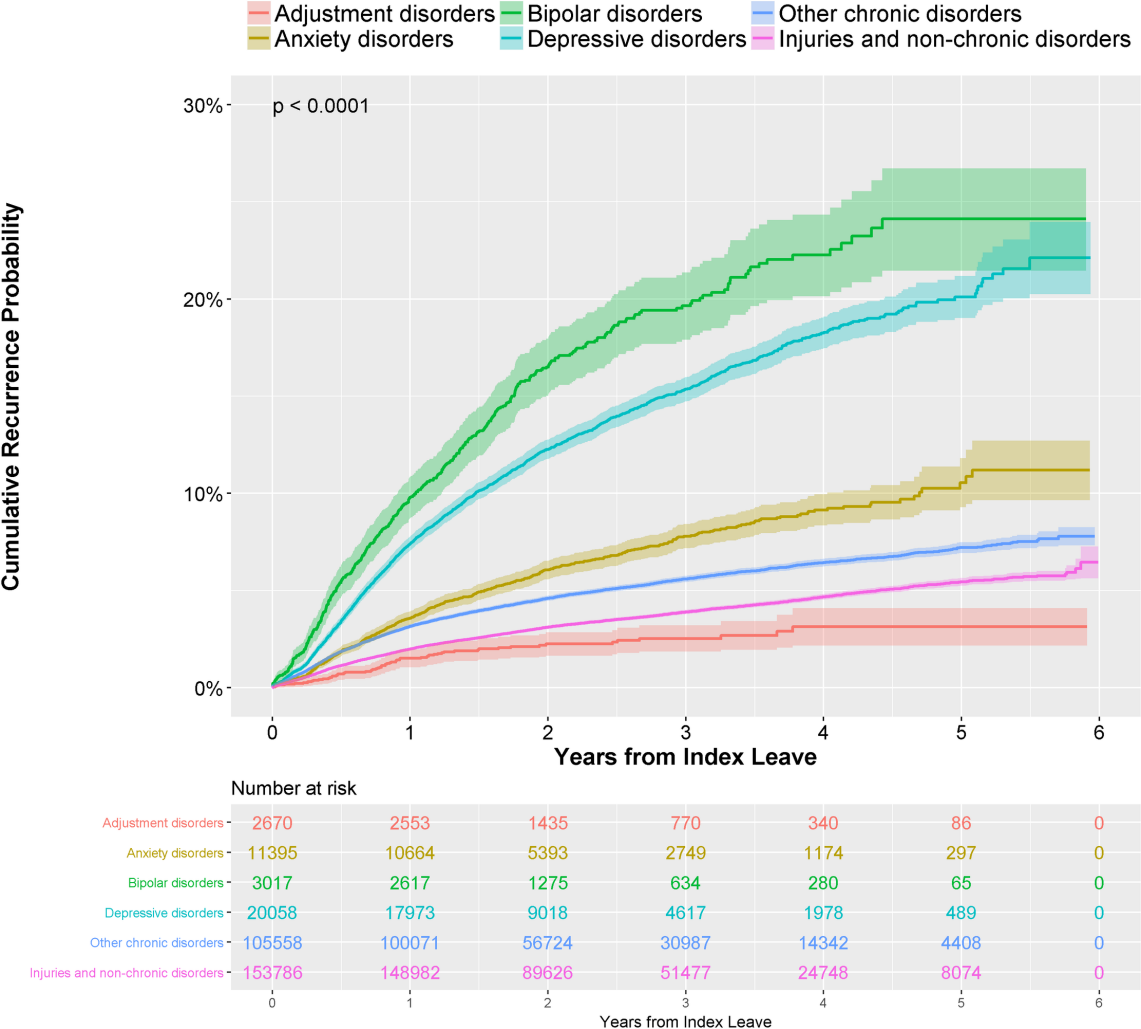
Kaplan-Meier curves representing time to recurrence, by condition.

Out of the four CMDs studied, bipolar disorder had both the highest probability of work disability recurrence within a year (9.7%) and recurrence density (98.7 per 1000 person-years), followed by depressive disorders (Table 2). The median time between the index and the first recurrent disability was always less than a year. The recurrent disability durations were typically longer than the index durations for all CMDs (Recurrence/Index Duration Ratio > 1). Compared to other chronic disorders and non-chronic disorders, the individuals with CMDs typically had greater recurrence probabilities within a year and recurrence densities. For example, the recurrence densities for bipolar and depressive disorders were more than three times greater than other chronic disorders (Fig 1). In addition, compared to individuals with other chronic disorders and non-chronic disorders, the individuals with CMDs had higher rates of disability recurrence for conditions distinct from their index leave. The median disability durations for bipolar and depressive disorders (57 and 50 days, respectively) were the longest of all conditions.

**Table 2:** Work disability summary statistics.

			Probability of recurrence within a year (%)		Recurrence density (# per 1000 person-years)		Median days until recurrence		
Condition	N	Median (IQR) duration of index leave^a^	Same disorder	Other disorder	Same disorder	Other disorder	Same disorder	Other disorder	Median recurrence/index duration ratio
Adjustment disorders	2,670	39 (24–70)	1.5	18.7	10.2	222.9	299	318	1.00
Anxiety disorders	11,395	37 (22–69)	3.6	21	32.4	262.4	316	290	1.13
Bipolar disorders	3,017	57 (32–93)	9.7	23.3	98.7	298.7	300	280	1.21
Depressive disorders	20,058	50 (29–87)	7.3	22	70.9	279.5	314	304	1.08
Other chronic disorders	105,558	43 (25–74)	3.1	14.6	23.3	165.9	265	338	1.08
Injuries or non-chronic disorder	153,786	34 (19–58)	2.0	9	15.2	94.3	314	383	1.06

^a^IQR = interquartile range

Using IPWs, work disability recurrence statistics were typically higher for the study population than the source population, but lower compared to the target population (Tables C-E in S1 File). For example, the probability of recurrence within a year for individuals with depressive disorders was 9.7% in our study population, but 8.4% and 10.4% in the source and target populations, respectively. Predictors of recurrence.

In univariate analyses, demographic risk factors for recurrent CMD-related disability included being female, younger, an hourly employee, in a union, and on disability longer for the index leave, although the associations do not hold for all four CMD groups (Table 3). Individuals with a previous, non-CMD leave had higher rates of CMD recurrence in all four CMD groups. Out of the geographically derived variables, only population density was a risk factor for recurrent depression disability. Employees in the transportation, communication or utilities industries were at greater risk for recurrence, whereas employees in the manufacturing of durable and non-durable goods industries were less at risk. Inpatient stays during the index leave were not associated with recurrence (p-value > 0.05). Point-of-service insurance coverage was a risk factor for recurrent work disability in individuals with anxiety and depressive disorders. The comorbidities noted in the year prior to the index leave that were risk factors for recurrent disability included depression, uncomplicated diabetes mellitus, obesity, psychoses, and chronic pulmonary disease.

**Table 3:** Results from univariate cox proportional hazard models.

	Adjustment disorders (n = 2,670)			Anxiety disorders (n = 11,395)			Bipolar disorders (n = 3,017)			Depression disorders (n = 20,058)		
Variable	HR^a^	95% CI^b^	p-value^c^	HR^a^	95% CI^b^	p-value^c^	HR^a^	95% CI^b^	p-value^c^	HR^a^	95% CI^b^	p-value^c^
**Sex (male = 1)**	1.01	0.60–1.70		0.92	0.79–1.07		0.90	0.75–1.08		0.80	0.74–0.88	***
**Age (years)**^**d**^	0.75	0.58–0.98	*	0.81	0.75–0.88	***	1.00	0.92–1.09		0.90	0.86–0.93	***
**Salaried (yes = 1)**	0.6	0.30–1.22		0.59	0.48–0.73	***	0.75	0.60–0.94	*	0.64	0.58–0.71	***
**Union (yes = 1)**	0.85	0.49–1.47		1.19	1.02–1.39	*	1.14	0.95–1.36		1.17	1.08–1.27	***
**Median household income**^**d**^^,^^**e**^	0.96	0.75–1.24		0.98	0.91–1.05		1.03	0.94–1.12		0.97	0.93–1.01	
**Percent of individuals with a college education or more**^**e**^	1.09	0.85–1.39		1	0.93–1.08		1.01	0.92–1.10		1.02	0.98–1.06	
**Population density (# per square mile)**^**d**^^,^^**e**^	1.12	0.91–1.38		1.03	0.96–1.10		1.01	0.93–1.10		1.07	1.04–1.11	***
**Previous non-CMD work leave prior to index leave (yes = 1)**	2.36	1.42–3.91	***	1.65	1.42–1.91	***	1.73	1.44–2.07	***	1.78	1.64–1.92	***
**Index duration (days)**^**d**^	1.14	0.97–1.33		1.11	1.05–1.18	***	1.00	0.91–1.09		1.08	1.04–1.11	***
**Inpatient stay during index leave (yes = 1)**	-	-	-	-	-	-	1.00	0.81–1.23		0.96	0.85–1.08	
**Number of outpatient psychiatric visits in the year prior to index leave**	1.06	0.86–1.31		1.12	1.07–1.18	***	1.19	1.11–1.27	***	1.21	1.18–1.24	***
**Employee in transportation, communication, utilities industries**	2.14	1.29–3.53	**	1.41	1.21–1.63	***	1.33	1.10–1.59	**	1.44	1.33–1.55	***
**Employee in manufacturing of durable goods industry**	0.39	0.18–0.86	*	0.81	0.67–0.98	*	0.93	0.76–1.13		0.90	0.81–0.99	*
**Employee in finance, insurance, real estate industries**	0.75	0.41–1.37		1.03	0.88–1.20		1.04	0.85–1.28		0.88	0.81–0.99	*
**Employee in manufacturing of non-durable goods industry**	0.91	0.36–2.27		0.52	0.36–0.74	***	0.59	0.41–0.87	**	0.66	0.54–0.80	***
**Consumer driven health plan**	-	-	-	0.96	0.68–1.36		-	-	-	0.79	0.64–0.97	*
**Health maintenance organization**	0.58	0.27–1.28		1.07	0.87–1.31		0.98	0.75–1.30		0.95	0.84–1.07	
**Point-of-service**	1.11	0.48–2.58		1.31	1.05–1.63	*	1.29	0.97–1.71		1.22	1.08–1.38	**
**Preferred provider organization**	1.22	0.71–2.10		0.91	0.78–1.06		0.95	0.78–1.15		1.03	0.95–1.12	
**Alcohol abuse**^**f**^	-	-	-	-	-	-	1.13	0.75–1.69		-	-	-
**Depression**^**f**^	1.15	0.69–1.94		1.30	1.11–1.52	**	1.23	1.03–1.46	*	1.50	1.38–1.62	***
**Diabetes mellitus, uncomplicated**^**f**^	1.00	0.36–2.75		0.68	0.48–0.96	*	0.91	0.65–1.27		1.05	0.91–1.21	
**Hypertension, uncomplicated**^**f**^	1.09	0.57–2.09		0.95	0.78–1.15		0.94	0.75–1.18		1.03	0.93–1.13	
**Hypothyroidism**^**f**^	1.78	0.77–4.13		1.06	0.80–1.40		1.03	0.75–1.41		1.05	0.91–1.22	
**Obesity**^**f**^	0.64	0.16–2.61		1.32	0.99–1.77		1.15	0.82–1.62		1.37	1.19–1.57	***
**Psychoses**^**f**^	-	-	-	-	-	-	1.46	1.09–1.94	*	-	-	-
**Chronic pulmonary disease**^**f**^	0.40	0.10–1.64		1.23	0.97–1.56		1.32	1.02–1.70	*	1.11	0.97–1.26	

^a^ Hazard ratio

^b^ 95% confidence interval

^c^ *** = p-value < 0.001; ** = p-value < 0.01; * = p-value < 0.05

^d^ Variable mean centered and scaled

^e^ Geographically-derived variables

^f^ Comorbidities present in year prior to index duration and defined by Quan et al. (2005)[18]

Variables not present or with insufficient variability in subset could not be tested and noted with”-“.

In the multiple variable regression analyses (Table 4), across the CMDs, the most robust predictors of reduced time to recurrent disability included having a non-CMD work leave before the index leave; being younger; working as an hourly employee; living in a more educated geographic area; having more outpatient visits; being an employee in the transportation, communications, and utilities industries; and being an employee in the finance, insurance, and real estate industries. The only significant risk factor of recurrent work disability due to adjustment disorders was having a previous non-CMD work leave prior to the index leave (hazard ratio = 2.36, 95% confidence interval = 1.42–3.91, p-value = 0.001).

**Table 4:** Results of multiple variable cox proportional hazard models.

	Anxiety disorders (n = 11,395)			Bipolar disorders (n = 3,017)			Depression disorders (n = 20,058)		
Variable	HR^a^	95% CI^b^	p-value	HR^a^	95% CI^b^	p-value	HR^a^	95% CI^b^	p-value
**Sex (male = 1)**	-	-	-	-	-	-	0.85	0.78–0.93	0.0001
**Age (years)** ^**c**^	0.83	0.77–0.90	<0.0001	-	-	-	0.92	0.88–0.96	<0.0001
**Salaried (yes = 1)**	0.70	0.57–0.86	0.0001	-	-	-	0.73	0.65–0.81	<0.0001
**Previous non-CMD leave prior to index leave (yes = 1)**	1.59	1.37–1.85	<0.0001	1.66	1.39–2.00	<0.0001	1.55	1.43–1.68	<0.0001
**Index duration (days)** ^**c**^	1.12	1.06–1.19	<0.0001	-	-	-	1.07	1.04–1.11	<0.0001
**Median household income**^**c**^^,^^**d**^	-	-	-	-	-	-	0.9	0.86–0.94	<0.0001
**Percent of individuals with college education or more**^**c**^^,^^**d**^	-	-	-	-	-	-	1.08	1.04–1.14	0.001
**Number of outpatient psychiatric visits in year prior to index leave** ^**c**^	1.11	1.06–1.17	<0.0001	1.16	1.08–1.24	<0.0001	1.16	1.13–1.20	<0.0001
**Employee in transportation, communication, utilities industries**^**e**^	1.44	1.19–1.74	<0.0001	1.38	1.13–1.70	0.002	1.7	1.45–1.98	<0.0001
**Employee in manufacturing of non-durable goods industry**^**e**^	-	-	-	-	-	-	1.35	1.14–1.59	0.0001
**Employee in finance, insurance, real estate industries**^**e**^	1.4	1.14–1.72	0.001	1.28	1.02–1.62	0.033	1.41	1.20–1.65	<0.0001
**Depression comorbidity noted in year prior to index leave**^**f**^	-	-	-	-	-	-	1.29	1.19–1.41	<0.0001

^a^ Hazard ratio

^b^ 95% confidence interval

^c^ Variable mean-centered and scaled

^d^ Geographically-derived variables

^e^ Reference group is employees in the other industries combined

^f^ Defined by Quan et al. (2005)[18]

Variables not selected as significant for a CMD noted with”-“.

In the univariate testing for effect modification by sex, the direction of association was predominantly the same for both male and females (Tables F and G in S1 File). Most significant variables were significant for both the males and female subsets, but never both significant and in opposite directions from the null. Further, no interaction variables were significantly associated with time to recurrence for any CMD in our multiple variable regression analyses (results not shown), suggesting no evidence for effect modification by sex.

## Discussion

For the first time, CMD recurrent disability statistics were calculated for a United States population. The probability and rate of recurrence for CMDs was typically higher than both other chronic disorders and injury/non-chronic disorders. Further, individuals with a CMD leave were more likely to go out on a subsequent disability for a condition distinct from their index leave. Demographic and employment variables were more likely to be associated with recurrent disability than comorbidities.

Recurrence metrics calculated in this study were generally consistent with those reported in the literature. For example, in a study on employees who worked in the Dutch Post and Telecommunications companies in the Netherlands, Koopmans et al. (2011) found a recurrence density for common mental disorders (CMD) (ICD-10 = R45, F32, F40, and F41) that ranged from 37.9 to 49.7 per 1000 person-years.[4] Whereas, recurrence densities in this study ranged from 10.2 to 98.7 per 1000 person-years. Also from the Netherlands, Norder et al. (2015) observed similar recurrence densities across the mental health conditions studied (ICD-10 = R45, F30-F49) in workers employed at a steel mill, with recurrence densities ranging from 29.9 to 37.7 per 1000 person-years.[7] A Brazilian study by Reis et al. (2011) appears to be an outlier with the authors reporting a recurrence density for mental and behavioral disorders (ICD-10 = F00–F99) of 6.7 per 100 worker-months or 806 per 1000 worker-years.[10] In this study, adjustment disorders appear to have different recurrence probability and rates compared to the other CMDs. Although adjustment disorders are considered chronic conditions, once the stressor or its consequences are removed, the symptoms of adjustment disorders should resolve within six months.[27,28] Therefore, the removal of stressors could protect against recurrence.

In a study of Canadian workers, Dewa et al. (2011) found that the median number of days between the end of the index leave and beginning of the recurrent leave for mental and behavior disorders (ICD-10 = F00-F99) was 673, which is approximately twice the time to recurrence for any CMD in this study.[29] However, results reported in this paper correspond with two studies from the Netherlands. Roelen et al. (2011) reported median days to recurrence for mental and behavioral disorders (ICD-10 = F00-F99) to be 326, and Koopmans et al. (2011) reported median months to recurrence for CMD (ICD-10 = R45, F32, F40, and F41) to be 10–11 months or 304–335 days.[4,6] In Koopmans et al. (2010), the authors reported that the index durations were typically longer than recurrent durations, which is opposite to the findings observed in this study.[30] If employees spend more time away from work during their subsequent leaves, this would indicate disease/condition progression and larger costs for employees and employers.

Interestingly, Koopman et al. (2011) had a disparate distribution of CMDs than our population. Excluding individuals with distress symptoms and other psychiatric disorders, as those groups were not specifically studied in this analysis, individuals with adjustment, depressive, and anxiety disorders accounted for 80%, 14%, and 6% of their population, respectively.[4] Those same groups accounted for 8%, 59%, and 33% of our population. It is unclear whether the difference can be attributed to diagnostic criteria or the population.

By identifying predictors of recurrent disability, our analyses could help identify the individuals who are more susceptible to recurrence so that programs can target additional care and disability management to them. In contrast to our findings, Norder et al. (2015) did not find that sex or whether an employee worked full or part-time to be related to recurrence rates. In this study, we observed this trend for depressive disorders, but not for the other CMDs; this is congruent with findings from Dewa et al. (2011). The higher rates of depression in females than males is an established difference.[31] In our study, being a salaried employee was a robust predictor of recurrent disability. This is in contrast to what was reported by Dewa et al. (2011). Future research should explore whether being a salaried employee is just a marker of socioeconomic status, benefits, or job type, as an individual’s socioeconomic position has been associated with recurrence.[12]

Norder et al. (2012) used a modified Delphi approach to establish a consensus opinion on the predictors of recurrent disability due to depression.[32] Their panel of both scientists and physicians believed the top predictors of recurrent disability for depression to be lifetime number of episodes, substance abuse, work dysfunction, and social dysfunction. Consistent with their consensus opinion, our results indicate that the number of previous outpatient psychiatric visits are related to recurrence. However, neither alcohol nor drug abuse comorbidities were associated with time to depression recurrence in our study.

Strengths of this research include the use of a nationwide database that provided a large number of potential determinants and statistical power. Survey weights were used to generate disability recurrence statistics to represent both the source and target populations. This research used the novel method of combining individual diagnoses into clinically meaningful categories using the CCS grouper, unlike previous research on CMD recurrence, which typically grouped diagnoses by ICD-10 code category (e.g., F32 for depression, and F43 for acute stress reactions and adjustment disorders) or the major diagnostic category. Further, calculating recurrence statistics for other chronic conditions and non-chronic conditions/injuries allowed for the disability experience of CMD patients to be compared with other conditions.

Limitations of the study include not knowing the exact dates of work disability eligibility, instead only knowing if an individual was eligible for the entire calendar year. However, it is reasonable to assume that an individual is just as likely to be eligible for disability benefits prior to the first full year of eligibility and after their last full year of eligibility. Therefore, although the exact eligibility dates were not known, this inaccuracy would be non-differential and would not bias the statistics in one direction. The requirement for individuals to have one year of STD eligibility without a CMD prior to their index leave may also have removed individuals with more frequent work disability.

Despite the use of survey weights, it is unclear if our recurrence statistics are representative of the United States population of employees with health and disability benefits. The MarketScan population is drawn from a convenience sample of employees with employer-provided health insurance and comes mostly from large employers, which may not generalize to the general population.[15] Given that we could only weight our recurrence statistics estimates using a limited set of covariates for the target population, it is important for future research to study work disability recurrence in other United States populations for comparison. Variables of potential interest that were missing from this analysis include ethnicity/race, educational attainment, the employers’ disability management protocols, individual salary and disability benefits information, and the type of work performed. Future disability recurrence research should attempt to use datasets with these variables available.

Future research should also explore differences in recurrence based on therapeutic and pharmacological interventions. In a cluster-randomized controlled trial of workers on sickness absence for CMD, where the intervention was a two-day training program in problem-solving, Arends et al. (2014) found the intervention group had a lower incidence of recurrent absences when compared with care as usual.[33] Further, Segal et al. (2010) found that cognitive therapy combined with pharmacotherapy was superior to antidepressant monotherapy for preventing depressive relapse.[34] In addition, future research should explore the work-related factors that contribute to work disability. For example, Prang et al. (2015) observed that the “mechanism” for the mental health condition including work pressure and harassment/bullying was significantly associated with multiple return-to-work attempts.[35]

There are several policy implications generated from this research. Given that individuals with CMD are more likely to have a work disability recurrence involving either their current CMD condition or another condition, CMD patients may benefit from additional care and disability management both during and after their work absence. These services may include increased provider or disability management touchpoints for individuals with CMD. Second, given that the duration of the recurrent leave was typically longer than the index leave, individuals with CMD are losing functional capacity through time. Health systems and leave management organizations should increase their focus on CMD disease management including sustaining treatment utilization.

## Conclusion

Individuals with CMD are more likely to have a recurrent disability than individuals with other chronic and non-chronic conditions. CMD disabilities may also make the individual more susceptible to work disability for conditions unrelated to their CMD. Therefore, CMD patients may benefit from additional care and disability management both during and after their work absence to help prevent recurrence of their CMD and non-CMD conditions.

## Supporting information

S1 Table ICD-9-CM codes assigned to disorder groups by healthcare cost and utilization project’s multi-level clinical classification software. (CSV)

S1 File Additional results. (DOCX)
