# Supplementary material for: Rates and predictors of recurrent work disability due to common mental health disorders in the United States
Source: PLoS One. 2018 Oct 9;13(10):e0205170. doi: 10.1371/journal.pone.0205170 (PMC6177162; doi:10.1371/journal.pone.0205170)
Supplement: S1 File — (DOCX) [file pone.0205170.s002.docx]

Table A. Comparison of demographic and occupational statistics by study, source, and target population.

|  | **Study Population,**  **n = 296,484** | **Source Population,**  **n = 8,806,801^1^** | **Target Population,**  **n =** **95,069,000^2^** |
| --- | --- | --- | --- |
| **Sex^3^** |  |  |  |
| Female | 51.3% | 56.1% | 53.1% |
| Male | 48.7% | 43.9% | 46.9% |
| **Industry^1,2^** |  |  |  |
| Construction, Agriculture, Forestry, Fishing | 0.1% | 0.1% | 8.2% |
| Finance, Insurance, Real Estate | 19.5% | 20.9% | 7.8% |
| Manufacturing, Durable Goods | 30.2% | 21% | 7.3% |
| Manufacturing, Nondurable Goods | 12.7% | 10.6% | 2.8% |
| Oil & Gas Extraction, Mining | 0.1% | 1.7% | 0.3% |
| Retail Trade | 4.4% | 7% | 10.3% |
| Services | 4.6% | 20% | 52.5% |
| Transportation, Communications, Utilities | 28.4% | 16.4% | 8.1% |
| Wholesale | <0.1% | 0.6% | 2.5% |
| Unknown or Other | <0.1% | 1.6% | 8.2% |
| **Salaried^1,4^** |  |  |  |
| No | 60% | 38.8% | NA |
| Yes | 25.4% | 39.5% | NA |
| Unknown | 14.6% | 21.7% | NA |
| **Union^1,5^** |  |  |  |
| No | 68.1% | 76.2% | 70.9% |
| Yes | 27.9% | 15.4% | 29.1% |
| Unknown | 4% | 8.3% | - |
| **Age^1,6^** |  |  |  |
| 18 to 24 years | 2.1% | 7.1% | 10.3% |
| 25 to 34 years | 18.5% | 26.2% | 24.4% |
| 35 to 44 years | 26.6% | 24.5% | 22.1% |
| 45 to 54 years | 32.3% | 24.1% | 23.7% |
| 55 to 65 years | 20.5% | 18.1% | 19.5% |
| **Health plan type^7^** |  |  |  |
| Consumer driven health plan | 6.6% | 7.4% | NA |
| Comprehensive | 2.4% | 1.8% | NA |
| Exclusive provider organization | 1.4% | 0.7% | NA |
| High-deductible health plan | 2.2% | 5.2% | 28% |
| Health maintenance organization | 11.3% | 9% | 14% |
| Point-of-service (POS) | 9.5% | 7.1% | 10% |
| POS with Capitation | <0.1% | 0.2% |  |
| Preferred provider organization | 66.2% | 46.6% | 48% |
| Missing/Other | 0.4% | 22% | 1% |

^1^ Source population is the entire Commercial Claims and Encounters database from 2008 to 2013, excluding individuals <18 years old. Includes individuals in the study population. For those not in the study population, the most recent demographic information available was used.

^2^ Target population is defined as an employed population, enrolled in employer-sponsored health care and disability benefits. Calculated using 2017 employed population statistics from the Bureau of Labor Statistics and health insurance information from the Kaiser Family Foundation.[1,2] Assumes individuals with health insurance have short-term disability insurance.

^3^ Target population estimates calculated from Bureau of Labor Statistics and the Kaiser Family Foundation [3,4]

^4^ Target population statistics not available. Although 52% of the employed population is hourly [5], no reliable estimates for percent of hourly workers with employer sponsored insurance were found.

^5^ Target population estimates calculated from Bureau of Labor Statistics.[6,7]

^6^ Target population’s age distributions from Bureau of Labor Statistics [8] and United States Census Bureau.[9]

^7^ Estimate are percentages across all firms by Kaiser Family Foundation.[2] Fewer health plan categories were obtained in the Kaiser Family Foundation study than Truven.

Table B. Population distributions used in raking procedure to develop inverse probability weights. Groups were combined to ensure study population cells were not less than 5%. [10]

|  | **Study Population,**  **n = 296,484** | **Target Population,**  **n =** **95,069,000** |
| --- | --- | --- |
| **Sex** |  |  |
| Female | 51.3% | 53.1% |
| Male | 48.7% | 46.9% |
| **Industry** |  |  |
| Finance, Insurance, Real Estate | 19.5% | 7.8% |
| Manufacturing, Durable Goods | 30.2% | 7.3% |
| Manufacturing, Nondurable Goods | 12.7% | 2.8% |
| Transportation, Communications, Utilities | 28.4% | 8.1% |
| Other | 9.1% | 74% |
| **Union** |  |  |
| No | 70.9% | 70.9% |
| Yes | 29.1% | 29.1% |
| **Age** |  |  |
| 18 to 34 years | 20.6% | 34.7% |
| 35 to 44 years | 26.6% | 22.1% |
| 45 to 54 years | 32.3% | 23.7% |
| 55 to 65 years | 20.5% | 19.5% |
| **Health plan type** |  |  |
| Consumer driven health plan, High-deductible health plan | 8.9% | 28% |
| Comprehensive, Exclusive provider organization, Health maintenance organization | 15.1% | 14% |
| Point-of-service (POS), POS with capitation | 9.6% | 10% |
| Preferred provider organization | 66.4% | 48% |

Table C. Comparison of disability durations and probability of work disability recurrence within the year by population.

|  | Median (IQR) days duration of index leave^a^ | | | Probability of recurrence within year (%)- same disorder | | | Probability of recurrence within year (%)- other disorder | | |
| --- | --- | --- | --- | --- | --- | --- | --- | --- | --- |
|  | **Study** | **Source** | **Target** | **Study** | **Source** | **Target** | **Study** | **Source** | **Target** |
| Adjustment disorders | 39 (24-70) | 42 (26-71) | 35 (22-68) | 1.5 | 1.5 | 2.0 | 18.7 | 15.3 | 21.2 |
| Anxiety disorders | 37 (22-69) | 40 (23-71) | 37 (21-73) | 3.6 | 3 | 4.1 | 21 | 16.8 | 22.3 |
| Bipolar disorders | 57 (32-93) | 59 (35-92) | 58 (31-99) | 9.7 | 8.4 | 10.4 | 23.3 | 19.1 | 21.7 |
| Depressive disorders | 50 (29-87) | 52 (30-87) | 51 (29-90) | 7.3 | 6.2 | 8.1 | 22 | 17.8 | 22 |
| Other chronic disorders | 43 (25-74) | 44 (26-73) | 41 (22-74) | 3.1 | 2.9 | 3.2 | 14.6 | 12 | 15.6 |
| Injuries or non-chronic disorder | 34 (19-58) | 35 (20-59) | 31 (16-57) | 2.0 | 1.9 | 2.0 | 9 | 7.7 | 9.5 |

^a^IQR = interquartile range

Table D. Comparison of work disability recurrence densities within the year by population.

|  | Recurrence density (# per 1000 person-years)- same disorder | | | Recurrence density (# per 1000 person-years)- other disorder | | |
| --- | --- | --- | --- | --- | --- | --- |
|  | **Study** | **Source** | **Target** | **Study** | **Source** | **Target** |
| Adjustment disorders | 10.2 | 9.5 | 15.6 | 222.9 | 169.5 | 250.4 |
| Anxiety disorders | 32.4 | 28.5 | 36.6 | 262.4 | 203.8 | 266.5 |
| Bipolar disorders | 98.7 | 83.4 | 106.8 | 298.7 | 222.5 | 277.6 |
| Depressive disorders | 70.9 | 58.5 | 85.5 | 279.5 | 221.3 | 271.6 |
| Other chronic disorders | 23.3 | 21.2 | 24.2 | 165.9 | 131.4 | 177.6 |
| Injuries or non-chronic disorder | 15.2 | 14.2 | 15.2 | 94.3 | 76.9 | 99.3 |

Table E. Comparison of time to work disability recurrence and recurrence/index duration by population.

|  | Median days until recurrence – same disorder | | | Median days until recurrence – other disorder | | | Median recurrence/index duration ratio | | |
| --- | --- | --- | --- | --- | --- | --- | --- | --- | --- |
|  | **Study** | **Source** | **Target** | **Study** | **Source** | **Target** | **Study** | **Source** | **Target** |
| Adjustment disorders | 299 | 310 | 289 | 318 | 344 | 319 | 1.00 | 0.99 | 0.97 |
| Anxiety disorders | 316 | 317 | 324 | 290 | 298 | 295 | 1.13 | 1.18 | 1.17 |
| Bipolar disorders | 300 | 275 | 308 | 280 | 266 | 305 | 1.21 | 1.23 | 1.22 |
| Depressive disorders | 314 | 305 | 340 | 304 | 310 | 314 | 1.08 | 1.09 | 1.08 |
| Other chronic disorders | 265 | 244 | 281 | 338 | 332 | 346 | 1.08 | 1.08 | 1.12 |
| Injuries or non-chronic disorder | 314 | 298 | 339 | 383 | 355 | 394 | 1.06 | 1.05 | 1.12 |

Table F. Results from univariate Cox proportional hazard models for adjustment and anxiety disorders, stratified by sex. Variables not present or with insufficient variability in subset could not be tested and noted with ”-“.

|  | Adjustment disorders | | | | | | Anxiety disorders | | | | | |
| --- | --- | --- | --- | --- | --- | --- | --- | --- | --- | --- | --- | --- |
|  | Males (n = 947) | | | Females (n = 1,723) | | | Males (n = 4,153) | | | Females (n = 7,242) | | |
| **Variable** | **HR^a^** | **95% CI^b^** | **p-value^c^** | **HR^a^** | **95% CI^b^** | **p-value^c^** | **HR^a^** | **95% CI^b^** | **p-value^c^** | **HR^a^** | **95% CI^b^** | **p-value^c^** |
| **Age (years)^d^** | 0.89 | 0.58-1.35 |  | 0.68 | 0.49-0.96 | * | 0.8 | 0.70-0.91 | *** | 0.82 | 0.75-0.91 | *** |
| **Salaried (yes = 1)** | 1.63 | 0.64-4.18 |  | 0.25 | 0.08-0.83 | * | 0.56 | 0.39-0.81 | ** | 0.6 | 0.47-0.77 | *** |
| **Union (yes = 1)** | 0.51 | 0.21-1.25 |  | 1.2 | 0.60-2.41 |  | 1.3 | 1.01-1.66 | * | 1.18 | 0.96-1.45 |  |
| **Median household income^d,e^** | 1.27 | 0.87-1.87 |  | 0.78 | 0.55-1.10 |  | 1.06 | 0.94-1.19 |  | 0.93 | 0.85-1.02 |  |
| **Percent of individuals with college education or more^e^** | 1.37 | 0.91-2.05 |  | 0.93 | 0.68-1.27 |  | 0.96 | 0.84-1.08 |  | 1.03 | 0.94-1.13 |  |
| **Population density (# per square mile)^d,e^** | 1.24 | 0.91-1.69 |  | 1.02 | 0.76-1.39 |  | 1.02 | 0.91-1.14 |  | 1.03 | 0.95-1.13 |  |
| **Previous non-CMD leave prior to index leave (yes = 1)** | 2.2 | 0.95-5.07 |  | 2.47 | 1.30-4.70 | ** | 1.61 | 1.26-2.07 | *** | 1.66 | 1.38-1.99 | *** |
| **Index duration (days)^d^** | 1 | 0.66-1.54 |  | 1.19 | 1.01-1.40 | * | 1.17 | 1.08-1.28 | *** | 1.07 | 0.99-1.16 |  |
| **Inpatient stay during index leave (yes = 1)** | - | - | - | - | - | - | 0.54 | 0.25-1.14 |  | - | - | - |
| **Number of outpatient psychiatric visits in year prior to index leave** | 1.14 | 0.84-1.55 |  | 1.01 | 0.74-1.37 |  | 1.14 | 1.04-1.24 | ** | 1.12 | 1.05-1.19 | *** |
| **Employee in transportation, communication, utilities industries^f^** | 2.46 | 1.06-5.69 | * | 1.96 | 1.05-3.67 | * | 1.39 | 1.08-1.78 | ** | 1.42 | 1.18-1.71 | *** |
| **Employee in manufacturing of durable goods industry^f^** | 0.63 | 0.25-1.61 |  | 0.12 | 0.02-0.90 | * | 0.81 | 0.61-1.07 |  | 0.83 | 0.63-1.10 |  |
| **Employee in finance, insurance, real estate industries^f^** | 1.29 | 0.44-3.82 |  | 0.61 | 0.30-1.25 |  | 1.14 | 0.83-1.57 |  | 0.97 | 0.80-1.17 |  |
| **Employee in manufacturing of non-durable goods industry^f^** | - | - | - | 1.63 | 0.64-4.17 |  | 0.53 | 0.31-0.91 | * | 0.51 | 0.31-0.82 | ** |
| **Consumer driven health plan** | - | - | - | - | - | - | - | - | - | 0.98 | 0.66-1.47 |  |
| **Health maintenance organization** | 0.6 | 0.18-2.04 |  | 0.57 | 0.20-1.60 |  | 1.13 | 0.82-1.56 |  | 1.04 | 0.79-1.37 |  |
| **Point-of-service** | 1.23 | 0.36-4.15 |  | 1.01 | 0.31-3.28 |  | 1.72 | 1.27-2.34 | *** | 1.04 | 0.75-1.43 |  |
| **Preferred provider organization** | 0.83 | 0.36-1.93 |  | 1.61 | 0.76-3.38 |  | 0.77 | 0.60-0.99 | * | 1 | 0.82-1.22 |  |
| **Depression^g^** | 1.78 | 0.77-4.12 |  | 0.89 | 0.46-1.74 |  | 1.15 | 0.86-1.52 |  | 1.37 | 1.13-1.66 | ** |
| **Diabetes mellitus, uncomplicated^g^** | 0.66 | 0.09-4.91 |  | 1.18 | 0.36-3.83 |  | 0.83 | 0.51-1.36 |  | 0.58 | 0.36-0.95 | * |
| **Hypertension, uncomplicated^g^** | 1.68 | 0.66-4.29 |  | 0.77 | 0.30-1.96 |  | 1.04 | 0.77-1.42 |  | 0.89 | 0.70-1.15 |  |
| **Hypothyroidism^g^** | - | - | - | 1.38 | 0.49-3.89 |  | - | - | - | 0.93 | 0.68-1.29 |  |
| **Obesity^g^** | - | - | - | 0.79 | 0.19-3.28 |  | - | - | - | 1.24 | 0.88-1.73 |  |
| **Chronic pulmonary disease^g^** | - | - | - | 0.56 | 0.13-2.31 |  | 1.49 | 0.99-2.24 |  | 1.11 | 0.82-1.50 |  |

^a^ Hazard ratio

^b^ 95% confidence interval

^c^ *** = p-value < 0.001; ** = p-value < 0.01; * = p-value < 0.05

^d^ Variable mean centered and scaled

^e^ Geographically-derived variables

^f^ Reference group is employees in the other industries combined

^g^ Comorbidities present in year prior to index duration and defined by Quan et al. (2005)[11]

Table G. Results from univariate Cox proportional hazard models for bipolar and depressive disorders, stratified by sex. Variables not present or with insufficient variability in subset could not be tested and noted with ”-“.

|  | Bipolar disorders | | | | | | Depressive disorders | | | | | |
| --- | --- | --- | --- | --- | --- | --- | --- | --- | --- | --- | --- | --- |
|  | Males (n = 1,177) | | | Females (n = 1,840) | | | Males (n = 6,217) | | | Females (n = 13,841) | | |
| **Variable** | **HR^a^** | **95% CI^b^** | **p-value^c^** | **HR^a^** | **95% CI^b^** | **p-value^c^** | **HR^a^** | **95% CI^b^** | **p-value^c^** | **HR^a^** | **95% CI^b^** | **p-value^c^** |
| **Age (years)^d^** | 0.98 | 0.85-1.13 |  | 1.02 | 0.91-1.14 |  | 0.99 | 0.92-1.07 |  | 0.87 | 0.83-0.91 | *** |
| **Salaried (yes = 1)** | 0.71 | 0.49-1.03 |  | 0.77 | 0.58-1.02 |  | 0.72 | 0.59-0.87 | *** | 0.61 | 0.54-0.68 | *** |
| **Union (yes = 1)** | 1.19 | 0.89-1.59 |  | 1.15 | 0.90-1.46 |  | 1.19 | 1.03-1.38 | * | 1.24 | 1.12-1.37 | *** |
| **Median household income^d,e^** | 0.95 | 0.82-1.10 |  | 1.08 | 0.97-1.20 |  | 1 | 0.93-1.08 |  | 0.96 | 0.92-1.00 |  |
| **Percent of individuals with college education or more^e^** | 0.93 | 0.81-1.08 |  | 1.06 | 0.95-1.19 |  | 1.02 | 0.95-1.10 |  | 1.02 | 0.98-1.07 |  |
| **Population density (# per square mile)^d,e^** | 1.11 | 0.98-1.25 |  | 0.95 | 0.84-1.07 |  | 1.11 | 1.04-1.18 | ** | 1.06 | 1.02-1.11 | ** |
| **Previous non-CMD leave prior to index leave (yes = 1)** | 1.44 | 1.08-1.93 | * | 1.94 | 1.54-2.44 | *** | 1.72 | 1.48-2.00 | *** | 1.78 | 1.62-1.95 | *** |
| **Index duration (days)^d^** | 0.95 | 0.81-1.12 |  | 1.03 | 0.93-1.16 |  | 1.1 | 1.04-1.16 | ** | 1.06 | 1.02-1.11 | ** |
| **Inpatient stay during index leave (yes = 1)** | 0.98 | 0.72-1.34 |  | 1.03 | 0.79-1.35 |  | 0.84 | 0.67-1.05 |  | 1.05 | 0.91-1.21 |  |
| **Number of outpatient psychiatric visits in the year prior to index leave** | 1.23 | 1.09-1.37 | *** | 1.17 | 1.08-1.28 | *** | 1.24 | 1.18-1.30 | *** | 1.2 | 1.16-1.24 | *** |
| **Employee in transportation, communication, utilities industries^f^** | 1.19 | 0.88-1.62 |  | 1.39 | 1.11-1.74 | ** | 1.17 | 1.00-1.36 | * | 1.54 | 1.41-1.69 | *** |
| **Employee in manufacturing of durable goods industry^f^** | 1.04 | 0.78-1.39 |  | 0.87 | 0.65-1.17 |  | 1.04 | 0.89-1.21 |  | 0.89 | 0.78-1.02 |  |
| **Employee in finance, insurance, real estate industries^f^** | 0.84 | 0.54-1.31 |  | 1.08 | 0.85-1.37 |  | 0.93 | 0.76-1.15 |  | 0.82 | 0.75-0.90 | *** |
| **Employee in manufacturing of non-durable goods industry^f^** | 0.72 | 0.42-1.24 |  | 0.51 | 0.30-0.87 | * | 0.65 | 0.48-0.90 | ** | 0.68 | 0.54-0.86 | ** |
| **Consumer driven health plan** | - | - | - | - | - | - | - | - | - | 0.69 | 0.54-0.89 | ** |
| **Health maintenance organization** | 0.89 | 0.59-1.36 |  | 1.09 | 0.75-1.57 |  | 0.92 | 0.74-1.14 |  | 0.99 | 0.86-1.15 |  |
| **Point-of-service** | 1.11 | 0.68-1.80 |  | 1.41 | 0.99-2.00 |  | 1.16 | 0.92-1.45 |  | 1.28 | 1.10-1.49 | ** |
| **Preferred provider organization** | 1.03 | 0.76-1.40 |  | 0.88 | 0.69-1.13 |  | 1.02 | 0.87-1.19 |  | 1.01 | 0.91-1.11 |  |
| **Alcohol abuse^g^** | 1.21 | 0.70-2.09 |  | - | - | - | - | - | - | - | - | - |
| **Depression^g^** | 1.19 | 0.89-1.59 |  | 1.24 | 0.99-1.56 |  | 1.52 | 1.31-1.77 | *** | 1.48 | 1.35-1.63 | *** |
| **Diabetes mellitus, uncomplicated^g^** | 0.96 | 0.58-1.57 |  | 0.89 | 0.57-1.38 |  | 1.27 | 1.00-1.61 | * | 0.96 | 0.81-1.15 |  |
| **Drug abuse^g^** | 1.62 | 0.97-2.70 |  | - | - | - | - | - | - | - | - | - |
| **Hypertension, uncomplicated^f^** | 1.02 | 0.72-1.43 |  | 0.9 | 0.67-1.22 |  | 1.16 | 0.98-1.37 |  | 0.99 | 0.88-1.11 |  |
| **Hypothyroidism^f^** | - | - | - | 1.09 | 0.77-1.54 |  | - | - | - | 0.99 | 0.84-1.17 |  |
| **Obesity^f^** | - | - | - | 1.1 | 0.75-1.62 |  | - | - | - | 1.27 | 1.09-1.48 | ** |
| **Psychoses^f^** | 1.47 | 0.98-2.21 |  | 1.52 | 1.01-2.28 | * | - | - | - | - | - | - |
| **Chronic pulmonary disease^f^** | 1.09 | 0.68-1.75 |  | 1.43 | 1.05-1.94 | * | 1.04 | 0.78-1.39 |  | 1.1 | 0.95-1.27 |  |

^a^ Hazard ratio

^b^ 95% confidence interval

^c^ *** = p-value < 0.001; ** = p-value < 0.01; * = p-value < 0.05

^d^ Variable mean centered and scaled

^e^ Geographically-derived variables

^f^ Reference group is employees in the other industries combined

^g^ Comorbidities present in year prior to index duration and defined by Quan et al. (2005)[11]

**References**

1. Bureau of Labor Statistics. Household data annual averages: employed persons by detailed industry, sex, race, and hispanic or latino ethnicity. Labor Force Statistics from the Current Population Survey. 2017 [Accessed 2018 Jun 5]. Available from: https://www.bls.gov/cps/cpsaat18.htm

2. Claxton G, Rae M, Long M, Damico A. Employer health benefits - 2017 annual survey. 2017; Available from: http://files.kff.org/attachment/Report-Employer-Health-Benefits-Annual-Survey-2017

3. Bureau of Labor Statistics. Household data annual averages: Employed persons by sex, occupation, class of worker, full- or part-time status, and race. Labor Force Statistics from the Current Population Survey. 2017 [Accessed 2018 Jun 6]. Available from: https://www.bls.gov/cps/cpsaat12.pdf

4. Kaiser Family Foundation. Kaiser Family Foundation analysis of 2017 ASEC supplement to the current population survey, U.S. census bureau. Menlo Park, CA; 2017.

5. Bureau of Labor Statistics. Household data annual averages: Wage and salary workers paid hourly rates with earnings at or below the prevailing federal minimum wage by selected characteristics. Labor Force Statistics from the Current Population Survey. 2017 [Accessed 2018 Jun 6]. Available from: https://www.bls.gov/cps/cpsaat44.pdf

6. Bureau of Labor Statistics. Household data annual averages: Union affiliation of employed wage and salary workers by selected characteristics. Labor Force Statistics from the Current Population Survey. 2017 [Accessed 2018 Jun 6]. Available from: https://www.bls.gov/cps/cpsaat40.pdf

7. Bureau of Labor Statistics. Employee benefits in the United States - March 2017. 2017;(March):1–19. Available from: https://www.bls.gov/news.release/pdf/ebs2.pdf

8. Bureau of Labor Statistics. Household data annual averages: Employed persons by detailed industry and age. Labor Force Statistics from the Current Population Survey. 2017 [Accessed 2018 Jun 6]. Available from: https://www.bls.gov/cps/cpsaat18b.pdf

9. Barnett JC, Berchick ER. Health insurance coverage in the United States: 2016. United States Census Bureau. 2017;(September):7–10. Available from: https://www.census.gov/content/dam/Census/library/publications/2017/demo/p60-260.pdf

10. Debell M, Krosnick J a. Computing weights for the American National Election Study survey data. ANES Technical Report series, no. nes012427. Ann Arbor, MI, and Palo Alto, CA; 2009. Available from: http://www.electionstudies.org/resources/papers/nes012427.pdf

11. Quan H, Sundararajan V, Halfon P, Fong A. Coding algorithms for defining comorbidities in ICD-9-CM and ICD-10 administrative data. 2005;43(11). Available from: https://www.ncbi.nlm.nih.gov/pubmed/16224307
